# Loss of MeCP2 causes subtle alteration in dendritic arborization of retinal ganglion cells

**DOI:** 10.1080/19768354.2021.1920459

**Published:** 2021-05-04

**Authors:** Wooje Lee, Ramesh Mariappan, Koushitak De, Takbum Ohn

**Affiliations:** a Department of Cellular & Molecular Medicine, College of Medicine, Chosun University, Gwangju, South Korea; b Department of Cell Biology and Human Anatomy, School of Medicine, University of California at Davis, Davis, CA, USA

**Keywords:** Methyl-CpG-binding protein, MeCP2, mouse retina, thy1-GFP, retina ganglion cells

## Abstract

Methyl-CpG-binding protein (MeCP2) is highly expressed in neurons. It plays an important role in the development of synapses and the formation of circuits in the central nervous system (CNS). Mutations in *MECP2* cause neurodevelopmental disorders and mental retardation in humans. Therefore, it has become important to determine the distribution and function of MeCP2 *in vivo*. The retina consists of three nuclear cell layers and two layers of synapses; neurons in each layer are connected to form fine circuits necessary for visual signal transduction. Using immunohistochemical analysis, we found that MeCP2 was expressed in all nuclear cell layers, with differences in the levels of MeCP2 expression observed among the layers. To understand the structural defects in the retina due to the loss of MeCP2, we sought to elucidate the organization of the retinal structure in the *Mecp2* knockout (KO) mouse. Overall, we found a normal retinal structure in *Mecp2* KO mice. However, because *Mecp2* mutations have a highly variable effect on neuronal architecture, we analyzed morphological changes in a subset of retinal ganglion cells of *Mecp2* KO mice. In Thy1-GFP mice crossed with *Mecp2* mutant mice, Sholl intersections analyses showed a subtle increase in number of intersections due to increased branching proximal to the soma in *Mecp2* KO mice. Our results demonstrate that the expression of MeCP2 and the effects of *Mecp2* mutations are highly specific to tissue and cell types.

## Introduction

MeCP2 is a methyl-CpG binding protein capable of suppressing gene expression by recruiting co-repressor complexes to different gene expression regulatory regions (Nan et al. 1996). Recent studies demonstrate that MeCP2 plays a role in regulating multiple transcriptional, post-transcriptional, and post-translational processes (Chen et al. 2003; Chahrour et al. 2008; Li et al. 2013; Lee et al. 2020). Mutations in *MECP2* cause the neurodevelopmental disorder Rett syndrome (RTT) in humans (Bienvenu et al. 2000). The representative RTT brain has been characterized as arrested at early developmental stages, with highly variable abnormalities (Christodoulou 2001; Zappella et al. 2003). Neuroanatomical studies in the *Mecp2* mutant mouse reveal changes in neuron maturation as well as in axonal and dendritic morphology, axonal mistargeting, poor synaptic formation, and misregulation of neurotransmission (Kishi and Macklis 2004; Smrt et al. 2007). Overall, these studies demonstrate that a loss of MeCP2 causes defects in the formation and maintenance of neuronal circuits.

Even though it is evident that *Mecp2* mutations cause abnormality in the nervous system, it is hard to characterize the cellular specific phenotype due to the complexity of in *vivo* tissue. For these reasons, comparative analysis from *Mecp2* mutations has revealed both overlapping and divergent effects in various cell types, including dendritic and axonal morphology, and dendritic spine density/spine morphology (Stuss et al. 2012; Lee et al. 2014). Since *Mecp2* mutations could contain any of these abnormalities in specific cell types, the use of precisely defined cellular subtypes is required in comparative analysis.

The vertebrate retina is highly organized, with multiple layers of nerve cell bodies and layers of synapses. The outer nuclear layer (ONL) is enriched with rod and cone cell bodies; the inner nuclear layer (INL) contains bipolar, horizontal, and amacrine cell bodies; and the ganglion cell layer (GCL) contains ganglion cell bodies and displaced amacrine cell bodies (Tian and Copenhagen 2003). In this study, we used the retina as a model to study neuronal circuit formation in a *Mecp2* mutation mouse because of the enriched neuron cell types and fine neuronal connections present.

In contrast to previous reports (Jain et al. 2010), we found that all of the retina cell layers express MeCP2, but at different levels in different layers. Upon characterization of the *Mecp2* KO mouse at the gross anatomy level, we found normal-sized eyes and a well-organized retinal structure. For further detailed assessment of the morphological changes in retinal ganglion cells (RGC), we crossed Thy1-GFP mice, which express GFP under the control of the Thy-1 promoter, with *Mecp2* KO mice. The data demonstrated that loss of MeCP2 results in subtle increases in dendritic branch density in RGCs. Overall, the result supports the view that the expression of MeCP2 and the effects of MeCP2 null are highly context-dependent *in vivo*.

## Materials and methods

Animals: Female heterozygotes with an MeCP2^tm1.1Bird^ mutation (Guy et al. 2001) were maintained on a 129/SvEv background (Tacoma). The MeCP2^tm1.1Bird^ were crossed with mice expressing GFP (Tg(Thy1-EGFP)MJrs/J) (Feng et al. 2000) in a subset of RGCs and bipolar cells. All mice were housed in an animal facility approved by the University of California Animal Care and Use Committee. All procedures described here are in accordance with the NIH animal use guidelines and institution-approved animal use protocols.

Preparation of Retina for Immunostaining: Mice were euthanized using CO_2_ and enucleated eyes were chilled in dry ice-cooled propane for 1 min. The eyes were transferred into vials containing 97% MeOH + 3% acetic acid and stored at −80°C for 48hr. The eyes were then transferred to −20°C and incubated for 24hr. Following this, the eyes were placed in 100% EtOH 30–60 min and embedded in paraffin.

Immunohistochemistry: For fluorescent immunohistochemistry, sections of 10 µm thickness were de-paraffinized and incubated in 5% horse serum and 1% Triton X-100 in phosphate buffered saline (PBS) for 1hr at room temperature. Afterwards, sections were incubated overnight with a primary antibody. The following primary antibodies were used: chicken anti-MeCP2 (1:1000, gift from Janine LaSalle), rabbit anti-pS421 MeCP2 (1:1000, Abgent), rabbit anti-pS80 MeCP2 (1:1000, Abgent), rabbit anti-PAX6 (1:1000, Covance), rabbit anti-Pcdh21 (1:1000, gift from Yoshihiro Yoshihara), and mouse anti-Rhodopsin (1:1000, Millipore). Antibodies were visualized with appropriate secondary antibodies conjugated with Cy3 or Cy2. Staining intensity was measured using ImageJ and statistically analyzed by using GraphPad InStat3.

Preparation of Retinas for GFP imaging: Eyes were enucleated and hemisected following euthanization of the mice. The cornea, iris, lens, and vitreous humor were removed from the eyes and immersed in PBS. The excised retinas were immersed in 4% PFA in PBS for 30 min at room temperature. The retinas were then stored overnight at 4°C in 30% sucrose in PBS. A rabbit GFP antibody (1:500, Molecular Probes) was used to enhance the GFP signal. Fixed retinas were washed in PBS, incubated for 24hr in primary antibodies with 0.1% Triton X-100 at 4°C, and washed again in PBS. Retinas were then incubated in secondary antibody (1:50) with 0.1% Triton X-100, flattened and mounted on Super-Frost Plus slides (Fisher).

Sholl analysis of RGC dendrites: Using the Bonfire program (Langhammer et al. 2010), the Sholl analyses were performed. For statistical analysis of the dendritic morphological change, a *t*-test between the WT and *Mecp2* KO group for all RG_A_ was performed using GraphPad InStat3.

Cell Nuclei Analysis: Toluidine blue-Staining of nuclei was performed in the retina to quantify differences in the thickness of nuclear layers. Images in Figure 3(A–F) were acquired with a microscope (Nikon, eclipse E800). Brightness and contrast were only slightly adjusted using Adobe Photoshop CS5. The thickness of cell nuclei in WT in Mecp2 KO was analyzed by *t-*test using GraphPad InStat3.

## Results

### MeCP2 is differentially expressed and phosphorylated in different cell types in the retina

**Figure 1.**
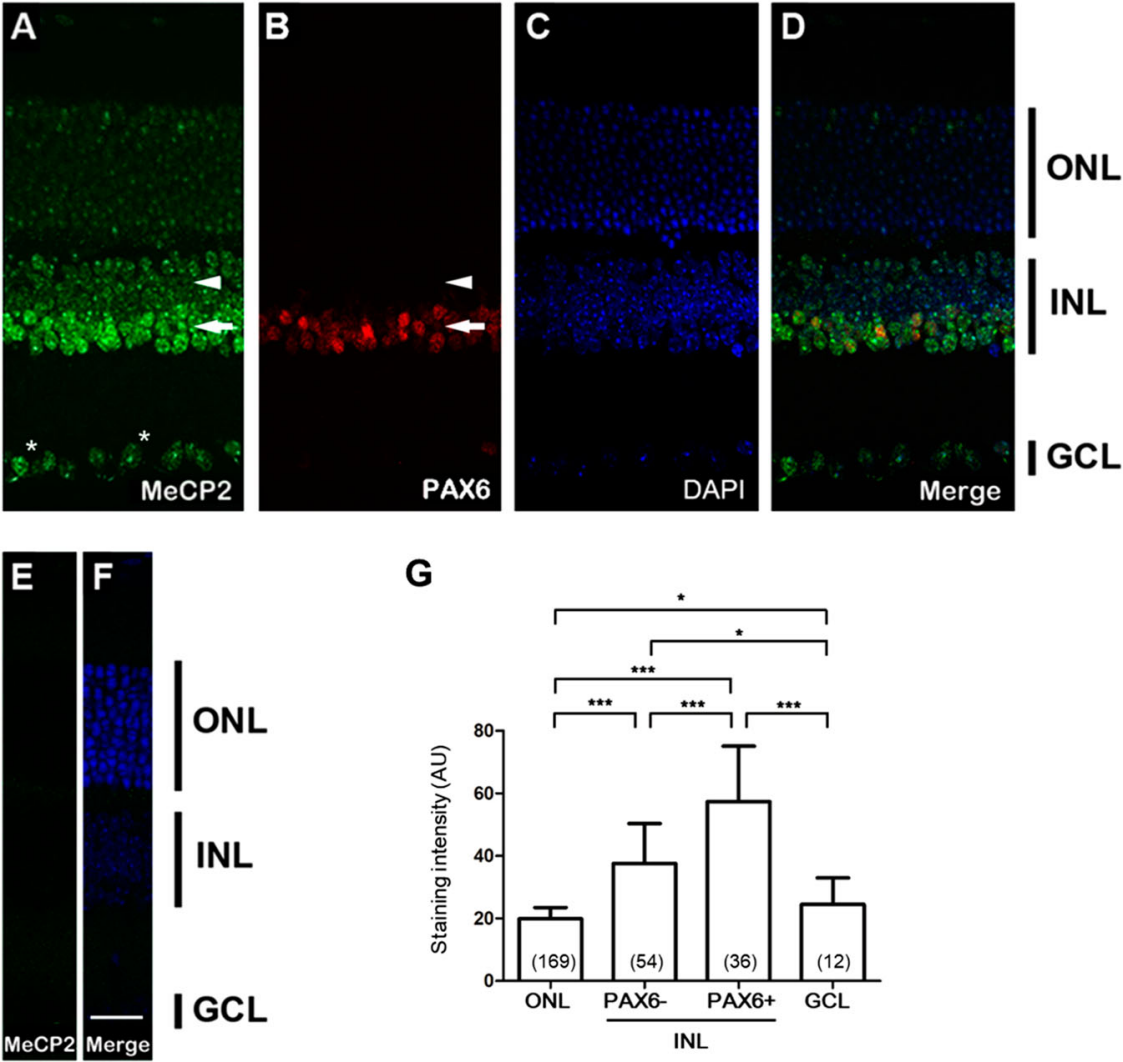
The retina of mouse showed a distinct MeCP2 expression pattern. (A) A C-terminal MeCP2 antibody was used to reveal the expression pattern of MeCP2 in the peripheral retina. (B) Pax6 labeled all amacrine cells. (C) DAPI stained all retinal nuclei of the outer nuclear layer (ONL), inner nuclear layer (INL), and ganglion cell layer (GCL). Pax6^+^ cells (arrow in B) express stronger levels of MeCP2 than Pax6^–^ cells (arrowhead in B). (E) With the same MeCP2 antibody, no visible signals were observed in the *Mecp2* KO mouse. (F) DAPI staining distinguished retinal nuclei of the ONL, INL, and GCL in *Mecp2* KO mice. Scale bar, 30 μm in all panels. (G) MeCP2 expression per each nucleus was quantified and statistically analyzed between the nuclear layer. *P < 0.05 and ***P < 0.001 by one-way ANOVA with Tukey’s post hoc test. Data are expressed as the mean ± SD. The number of nuclei for quantification is shown on each bar graph.

We performed localization studies of MeCP2 in the retina of postnatal WT mouse. Sagittal slices through the retina of 4-week-old male mice were stained for MeCP2 (Figure 1(A)), with a DAPI counterstain (Figure 1(C)) to visualize heterochromatin. MeCP2 was expressed exclusively within the nucleus of all cells. However, different levels of MeCP2 expression were observed between the ONL, INL, and GCL. In the ONL, DAPI (Figure 1(C)) staining showed a highly condensed pattern in the center of the photoreceptor cell nucleus. However, MeCP2 staining showed a relatively low and diffuse expression pattern (Figure 1(A)). In the INL, MeCP2 is highly co-localized with DAPI-positive heterochromatic foci. But, the levels of MeCP2 expression differed when comparing the upper cell layer (arrowhead in Figure 1(A)) and lower cell layer (arrow in Figure 1(A)) of the INL. To enable more detailed analysis, we used double immunolabeling for MeCP2 and Pax6 (Figure 1(B)). Strong Pax6 immunoreactivity was present in a subpopulation of the INL, amacrine, and horizontal cells (Hatakeyama and Kageyama 2004). The co-staining showed that most Pax6^+^ cells (arrow in Figure 1(B)) express significantly stronger levels of MeCP2 than Pax6^-^ cells (arrowhead in Figure 1(B)) at the INL (Figure 1(G)). The results suggest that MeCP2 has different levels of expression in different cell types within the INL. Immunostaining of MeCP2 and DNA (DAPI) in the GCL confirms the co-localization of MeCP2 and DAPI in neuronal nuclei (Figure 1(A,C)). The speckled nuclear staining of MeCP2 coincides with DAPI foci, but dispersed nuclear MeCP2 staining is reproducibly seen in a subpopulation of RGCs (star in Figure 1(A)). Overall, MeCP2 is significantly more expressed in INL and GCL than in ONL (Figure 1(G)). This observation is consistent with previous report (Song et al. 2014).

**Figure 2.**
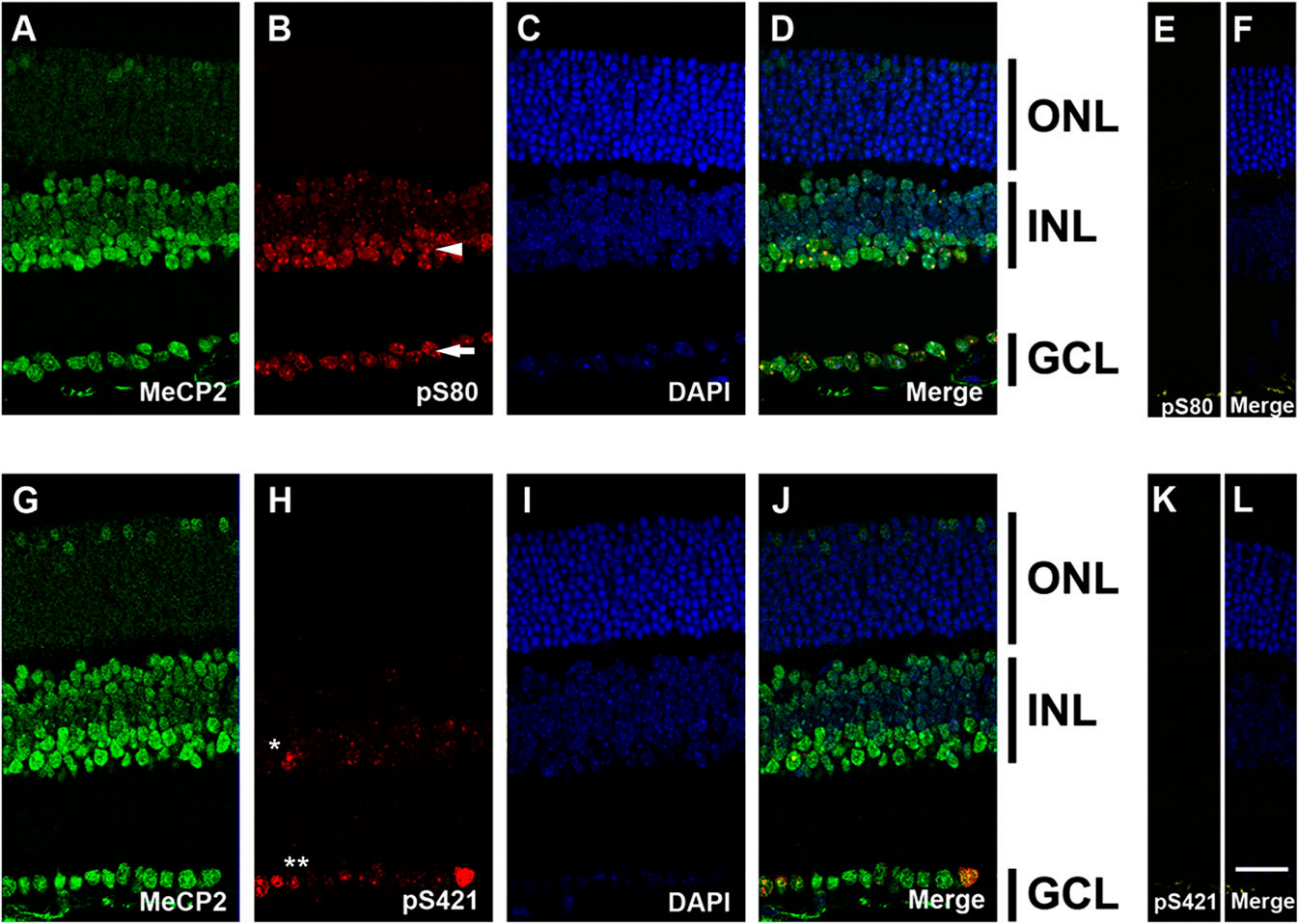
Subcellular localization of phosphorylated MeCP2 was observed in the retina. MeCP2 expression and different subcellular localization of phosphorylated MeCP2 are observed in retina. (A and G) C-terminal MeCP2 antibody showed that MeCP2 was expressed in the entire nuclear cell layer of retina. Phosphorylated MeCP2 at S80 (B) and at S421 (H) were visualized at in a subpopulation of cells. DAPI staining (C and I) was used as a counterstain. Strong levels of pS80 MeCP2 staining were observed in the lower cell layer of INL and GCL (arrowhead and arrow in B). Only limited cells contain phosphorylated MeCP2 at pS421 (star and double stars in H). (E and K) There was no expression of S80 and S421 in the *Mecp2* KO mouse. (F and L) DAPI distinguished the retinal nuclei of the ONL, INL, and GCL in *Mecp2* KO mouse. Scale bar, 30 μm in all panels.

It has been revealed that the dynamic balance between site-specific phosphorylation and dephosphorylation of MeCP2 controls the transcription of specific target genes (Zhou et al. 2006; Tao et al. 2009). In order to explore the subcellular localization of pS80, we used double immunostaining for MeCP2 (Figure 2(A)) with a pS80 MeCP2 specific antibody (Figure 2(B,E)). Interestingly, while weak MeCP2 staining was observed in all DAPI-positive heterochromatic chromocenters, no pS80 MeCP2 staining was detected in the ONL. In the lower cell layer of INL and GCL (arrowhead and arrow in Figure 2(B)), pS80 MeCP2 staining showed a punctate distribution within the heterochromatic region of nuclei. Consequently, we tested for MeCP2 phosphorylation localization at S421 (Figure 2(H,K)). While no visible signals of MeCP2 pS421 were observed in the ONL and the upper layer of the INL, limited cells in the lower layer of INL and GCL showed a punctate distribution of pS421 (star and double stars in Figure 2(H)). In the *Mecp2* KO mouse, there was no visible signals detected for MeCP2, pS80, or pS421, demonstrating the specificity of the antibodies (Figure 1(E) and Figure 2(E,K)). In addition, it is known that neuronal activity leads to MeCP2 phosphorylation at S421 (Zhou et al. 2006). Therefore, we compared the intensity and population of MeCP2 pS421-labelled cells between control mice and mice reared in the dark (24hr). No significant change was observed between these mice (data not shown). Taken together, MeCP2 is phosphorylated at pS80 in most nuclei and phosphorylation at pS421 is cell specific.

### Loss of MeCP2 causes limited impairment in retina structure

**Figure 3.**
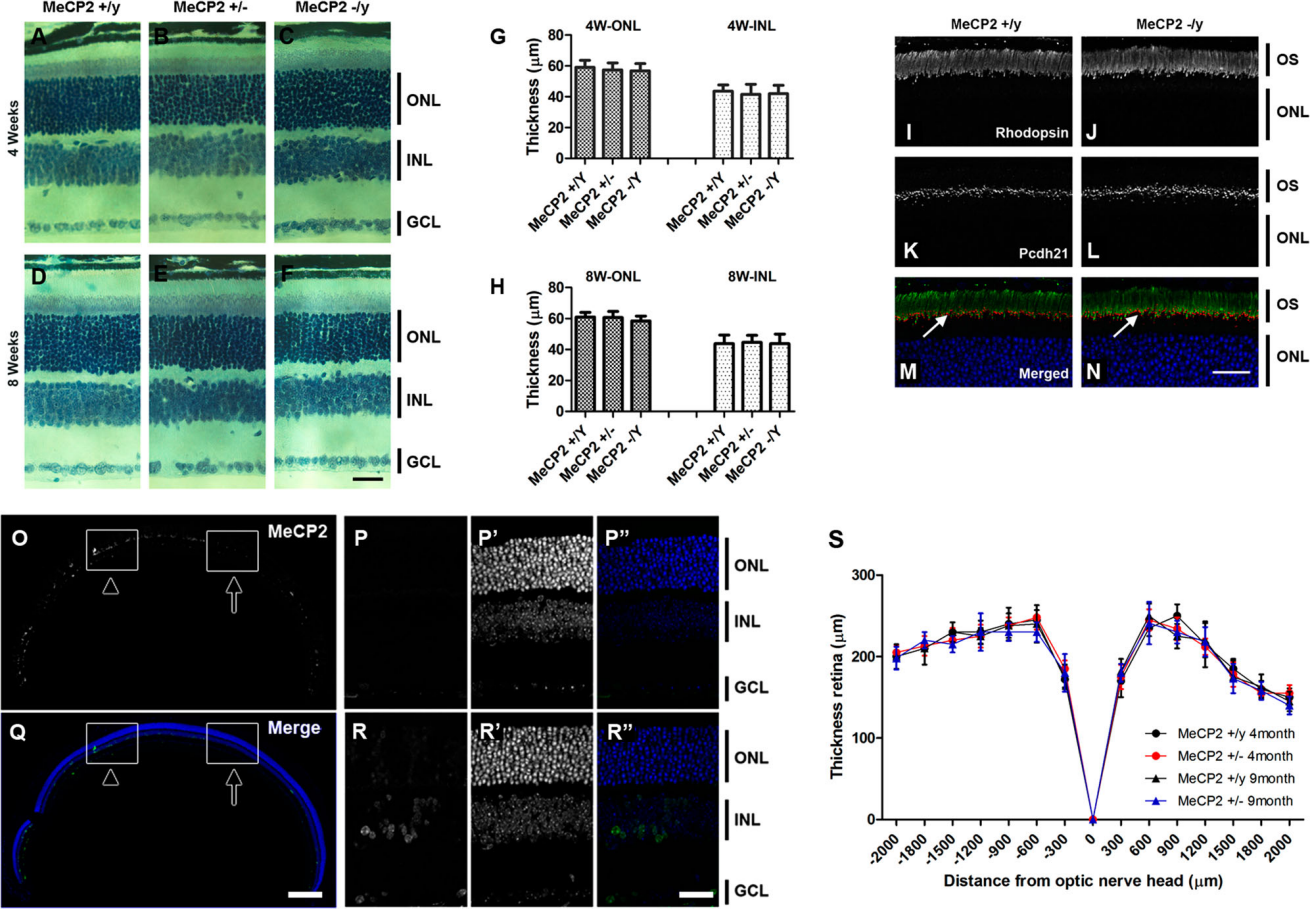
Gross morphometric analysis showed normal retinal structure in the adult *Mecp2* KO mouse. (A–F) Retina sagittal sections stained with toluidine blue visualized the ONL, INL, and GCL. Retina layer structure of WT, heterozygous, and KO mice were compared at 4 weeks (A–C) and 8 weeks (D–F). (G and H) The thickness of the ONL and INL area are compared at 4 and 8 weeks. Co-staining of Rhodopsin (I and J) and Pcdh21 (K and L) in the retina of WT and KO mice. (O and Q) Low magnification image of MeCP2 immunofluorescence showed a broad absence of MeCP2 staining in nuclei from the retina of *Mecp2* heterozygous mice. Scale bar, 200μm in O and Q. The regional MeCP2 expression of *Mecp2* heterozygous mice are shown at high magnification (P-P” and R-R”). Scale bar, 30μm in A-F, I-N, P-P” and R-R”. (S) Thickness from the optic nerve head was measured at 0, 300, 600, 900, 1200, 1500, 1800, and 2000 μm. Shown are the mean ± standard deviation of retinal thickness; *n* = 5.

Various studies have been conducted on Rett syndrome, but little is known about the retina. Therefore, we compared the thickness of nuclei in the ONL of animals aged 4 and 8 weeks, with the rationale that *Mecp2* deletion causes a degeneration of the ONL and INL. At 4 weeks (Figure 3(A–C,G)), the thickness (59.0 ± 4.64 μm, *n* = 5) of *Mecp2* KO ONL was 96.27% that of control (56.8 ± 4.66 μm, *n* = 5) (*p* = 0.61), and 95.7% at 8 weeks (WT; 61.0 ± 2.92 μm, *n* = 5 vs *Mecp2* KO; 58.40 ± 3.20 μm, *n* = 5) (*p* = 0.39) (Figure 3(D–F,H)). Overall, the thickness of cells in the ONL of *Mecp2* KO retina did not change and was statistically identical to that of WT littermates. We also sought to determine whether loss of MeCP2 in the INL affects the survival of INL cells. Under light microscopy, the thickness of the INL in *Mecp2* KO retinas was not obviously different from WT littermates at 4weeks (WT; 43.6 ± 1.81 μm, *n* = 5 vs *Mecp2* KO; 42.0 ± 2.43 μm, *n* = 5) (*p* = 0.61) and at 8weeks (WT; 43.8 ± 5.59 μm, *n* = 5 vs *Mecp2* KO; 43.8 ± 2.75 μm, *n* = 5) (*p* = 1.0) (Figure 3(A–H)). These data strongly suggest that overall cell viability is not affected in the *Mecp2* KO mouse.

The retina consists of multiple layers and has a sophisticated morphology. Misregulation of gene expression can often cause morphological changes in retina structure (Rattner et al. 2001). To determine whether primary cilium formation is defective in the absence of MeCP2, we compared the retinas of WT and *Mecp2* KO at 8 weeks. We performed immunostaining for Pcdh21 (Figure 3(K,L)), expressed at the interface between the retina and retinal pigment epithelium (RPE) (Rattner et al. 2001), and rhodopsin, which is most abundantly expressed in the outer segment (OS) (Figure 3(I,J)). Similar expression of Pcdh21 and rhodopsin was observed in retina sagittal sections of *Mecp2* KO. The OS of the photoreceptors appeared intact at the outer edge of the ONL in *Mecp2* KO when compared to WT. Therefore, we concluded that loss of MeCP2 does not cause impairment in the development of the photoreceptor outer segments.

### MeCP2 expression in *Mecp2* heterozygous mice

As *Mecp2* is located on the X chromosome, *Mecp2* heterozygous mice have a mosaic expression of MeCP2. Although *Mecp2* heterozygous mice should show random X chromosome inactivation (XCI) ratios, skewed XCI is observed in the heterozygous mouse (Braunschweig et al. 2004). In order to analyze the MeCP2 expression pattern in *Mecp2* heterozygous mice, sagittal sections through the retina were immunostained with C-terminal anti-MeCP2. A mosaic pattern of MeCP2 expression was observed in the retina nuclear layer (INL and ONL) in *Mecp2* heterozygous mice (Figure 3(O,Q)). However, the mean percentage of MeCP2-negative cells ranged from 20 ± 4.26% to 39.2 ± 5.23% in *Mecp2* heterozygous mice (*n* = 5), and this was lower than the overall expected 50%. In addition, there was some variation in the percentage of MeCP2-negative cells in the region of the retinal nuclear layer. For example, some regions exhibit mosaic expression (arrowhead in Figure 3(O,Q, and R-R”)), while others have little MeCP2 expression (arrow in Figure 3(O,Q, and P-P”)). These results indicate patchy regional distribution of MeCP2-expressing cells in the retina of *Mecp2* heterozygous mouse.

Due to the localization of the MeCP2 gene in the X chromosome, girls with *Rett* syndrome carry the defective gene in a portion of their retina for their entire lifetime. This impaired neuronal system is susceptible to degenerative disorders in old age. Here, we compared structural changes in the retina of *Mecp2* +/– for aged mouse (4 month and 9 month) with control littermates (Figure 3(S)). Histological experiment-based statistical analysis of retinas from *Mecp2* heterozygous mice revealed the absence of retinal degeneration in Mecp2 +/– and this was evident up to 9 months (Figure 3(S)). Taken together, these results demonstrate that MeCP2 was not necessary for structural maintenance.

### Retinal ganglion cells from *Mecp2* KO mice have morphological abnormalities in dendrites

To characterize the effects of MeCP2 loss on individual RGCs, we took advantage of the M-line of Thy1-GFP transgenic mice, which express GFP in a subpopulation of RGCs (Feng et al. 2000). We crossed homozygous Thy1-GFP-M transgenic mice with *Mecp2* heterozygous mice. GFP labeled RGCs, from the retinas of the three *Mecp2* WT/Thy1-GFP mice and three *Mecp2* KO/Thy1-GFP mice, were visualized with confocal microscopy. By using the organization of names provided by Sun et al. (2002) (Sun et al. 2002), 8RGCs evidently classified as RG_A_ were used for statistical analyses to demonstrate the dendritic morphological difference between WT and *Mecp2* KO.

**Figure 4.**
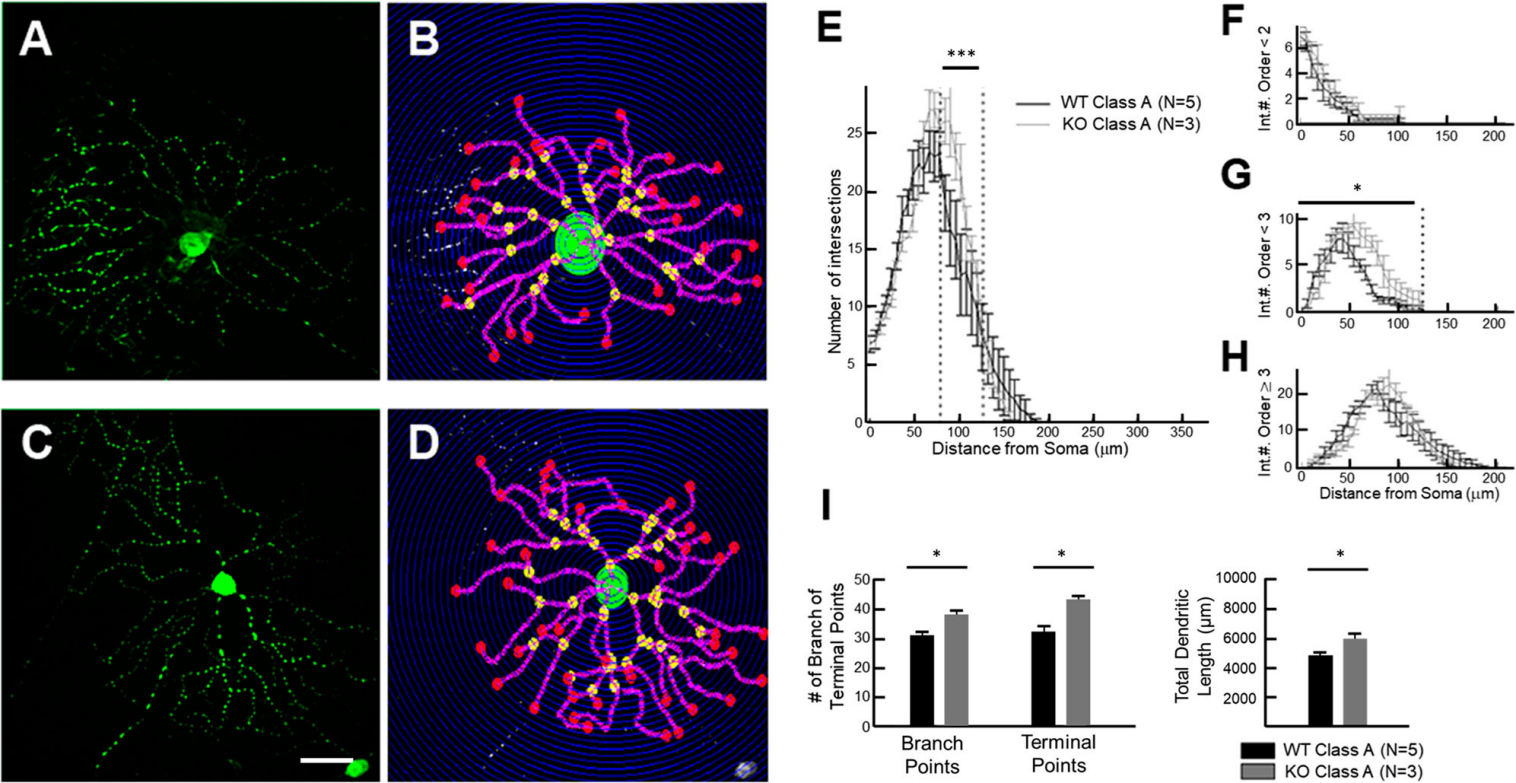
Dendritic arbor of retinal ganglion cells was visualized with GFP in Thy1-GFP labeled mouse. (A and C) Type RG_A_ from the retina of WT and *Mecp2* KO. (B and D) Reconstructions of (A) and (C) based on the Sholl analysis branch patterns. Dendrite density of Thy1-GFP RGCs was measured by assigning concentric circles, spaced at 7 µm intervals and centered on the soma. Retina from *Mecp2* KO/Thy1-GFP mice (4-week-old males) was stained with anti-GFP and counterstained with DAPI. Scale bar, 30μm in all panels. (E) The number of dendrites intersecting with concentric circles around the soma of each cell was analyzed. (F, G, and H) Sholl analysis, with segments grouped as either primary (F), secondary (G), or tertiary and greater (H). (I) Mean number of branch points, terminal points and total dendritic length was compared in RGCs of WT and *Mecp2* KO retina. Data are expressed as the mean ± SD.

Dendrite morphology was analyzed using a Sholl analysis program, Bonfire (Figure 4(A,B); RG_A_ in WT, C and D; RG_A_ in *Mecp2* KO) (Langhammer et al. 2010). In order to determine RGC changes in dendritic morphology between WT and *Mecp2* KO, we carried out a thorough morphometric analysis. The number of intersections gives a quantitative representation of how neurite density varies spatially. Statistical analysis revealed a significant change between 80 and 125 µm in the soma: roughly a 7.7% increase in number of intersections (Figure 4(E)). A more detailed picture of morphological change was generated using local-level Sholl analysis (Figure 4(F–H)). This analysis identified that the increased number of Sholl intersections was due to an increase in the number of secondary neurites (Figure 4(G)), causing a significant increase in the number of branch points up to 22.8% (*p* < 0.05) and up to 15.15% (*p* < 0.05) at terminal points (branch points WT; 26.65 ± 1.485, *n* = 5 vs *Mecp2* KO; 34.50 ± 1.397, *n* = 3, terminal points WT; 34.12 ± 1.35, *n* = 5 vs *Mecp2* KO; 40.21 ± 0.98, *n* = 3) (Figure 4(I)). Also, a significant increase was observed in total dendritic length up to 22.9% (*p* < 0.05, WT; 4720 ± 438.2, *n* = 5 vs *Mecp2* KO; 5800 ± 700 µm, *n* = 3) (Figure 4(I)). Overall, the morphometric parameters demonstrate more complexity in *Mecp2* KO retinas, when compared with the same types of RGCs from adult Thy1-GFP-M mice. We concluded that *Mecp2* KO RGCs retain their characteristic morphology, but have finer dendritic geometry.

## Discussion

The retina is a highly organized nervous system with several layers of nerve cell bodies and layers of synapses. Given that MeCP2 tends to be highly expressed in neuronal cell types (Kishi and Macklis 2004), the retina will be a suitable model for exploring the role of MeCP2 *in vivo*. MeCP2 expression was analyzed in detail during the development of retinal structures (Song et al. 2014). Specifically, MeCP2 begins to be expressed at E17 stage in ganglion and amacrine cells, whereas at P6 in bipolar cells and at 2 weeks of age in rod cells. In this study, we examined MeCP2 expression in the retina and whether the loss of MeCP2 leads to a neuropathological phenotype. We found that, among the three nuclear cell layers of the retina, the ONL had relatively low levels of MeCP2 expression, whereas MeCP2 was highly expressed in the cells of the INL and GCL. Using MeCP2 phosphorylation specific antibodies (Zhou et al. 2006; Tao et al. 2009), we found that pS80 MeCP2 was highly co-localized with pan-MeCP2 in the INL and GCL, pS421 MeCP2 was only observed in a subpopulation of cells in the INL and GCL. It has been reported that pS421 MeCP2 is increased in the suprachiasmatic nucleus (SCN) after light exposure (Zhou et al. 2006). However, light-induced neuronal activity did not increase MeCP2 phosphorylation at S421 in the retina. These results suggest that the level of MeCP2 expression varies from cell to cell in the retina, and that the signaling pathway that regulates MeCP2 phosphorylation in SCN may differ in the retina.

Cell subtypes were visualized at the single cell level using the Thy1-GFP line, and precisely defined subtypes of RGC were analyzed for dendritic morphology changes in *Mecp2* KO. Loss of MeCP2 induced significant but residual levels of augmented branching in the dendrites of RGCs. The data presented here support and extend similar findings that have recently been reported in *Mecp2* KO (Kishi and Macklis 2004; Fukuda et al. 2005; Stuss et al. 2012; Lee et al. 2014). However, we need to take a cautious approach in interpreting the results, because the effects of MeCP2 null are mostly cell type- or tissue type-dependent. For example, while mitral cell dendrites developed normally in *Mecp2* KO (Palmer et al. 2012), pyramidal neurons in the motor cortex showed deficient arborization (Stuss et al. 2012). Recent studies have shown that MeCP2 regulates the gene expression through binding to methylated DNA as well as various epigenetic modifications (Mellén et al. 2012; Lee et al. 2020). Considering that different cells have different epigenetic modifications, it suggests that morphological changes in *Mecp2* mutants may be caused by cell-specific mechanisms.

As a part of the mechanism to explain the morphological changes, it was reported that *Bdnf* gene expression is suppressed by MeCP2 binding to the promoter region of the *Bdnf* gene (Zhou et al. 2006). In *Mecp2* KO, the absence of MeCP2 triggers overexpression of the *Bdnf* gene and causes morphological changes in dendritic / axonal morphology, dendritic spine density, synapse formation and maturation through secondary neuronal structure modulation (Kishi and Macklis 2004; Fukuda et al. 2005; Smrt et al. 2007). However, genome-wide analysis studies showed that, in the absence of *Mecp2*, a broad number of genes are significantly affected including transcription- and translation regulatory genes (Chahrour et al. 2008; Li et al. 2013). Here, it is highly likely that other altered gene expressions exacerbated or compensated for morphological or physiological changes.

Various defects have been observed in RTT patients and *Mecp2* null mice, but so far few have been reported to have ocular defects in RTT patients (Jain et al. 2010; Townend et al. 2018; de Breet et al. 2019). This study detailed MeCP2 expression in the retina and analyzed structural changes including ganglion cell morphology in the retina of *Mecp2* KO. Different cells in the retina exhibit different levels of MeCP2 expression and intranuclear distribution. Therefore, it is necessary to analyze the role of MeCP2 at the single cell level for an in-depth understanding.
